# Fears during the Covid-19 pandemics and their influence on physical health: A cross-sectional study on the general population in Spain

**DOI:** 10.1016/j.ijchp.2022.100361

**Published:** 2022-11-24

**Authors:** José M. Peiró, Aina Luque-García, Aida Soriano, Vicente Martínez-Tur

**Affiliations:** a IDOCAL, Universitat de València, Spain; b IVIE, Spain; c Universidad Europea de Valencia, Spain

**Keywords:** Covid-19 fears, Rumination, Psychological distress, Somatic problems

## Abstract

**Background/objective:**

This study examines the paths through which Covid-19 can negatively impact health and lead to somatic symptoms. Based on the dual process theory, fears can impair health in two ways: through psychological distress, which is an automatic reaction to fear, and through a more conscious and deliberative rumination process.

**Method:**

Data from a representative sample of the Spanish population (*N* = 3083 subjects,18 years or older) were obtained from a Survey by the Sociological Research Center (CIS). The dual path model was tested, and a longer sequence was included where the two mediators act sequentially to produce an impact on somatic symptoms.

**Results:**

The results showed how Covid-19 fears translate into somatic problems. Beyond the direct relations, and after comparing with other possible alternative models, our findings support a process where rumination mediates between fears and psychological distress, and psychological distress in turn leads to somatic problems.

**Conclusions:**

This process reveals a plausible mechanism that explains the somatization of health problems during the Covid-19 pandemic, and it provides theoretical and practical inputs to better understand the role of fears in health in crisis contexts.

## Introduction

The global Covid-2019 pandemic continues to be a health threat, and it has produced disruptive alterations in health systems (Bueno-Notivol, et al., 2021; Shevlin et al., 2020), the economy, business, work activities, family life, and personal and social habits (Mertens et al., 2020). As a result, it has been found to have an impact on psychological and physical health (Sandín et al., 2020). In this situation, fear is one of the critical emotions that humans experience, and it can play a functional and adaptive role or have a dysfunctional and damaging effect on health and wellbeing (García, 2017; Green & Witte, 2006; Luo et al., 2021; Morman, 2000; Shevlin et al., 2020). Thus, it is important to understand how pandemic fear affects individual health and wellbeing.

Additionally, it is important to consider the dynamic features of the pandemic and related issues (e.g., lockdown period, vaccine availability, etc.) (Zhang et al., 2021). Most of the studies published so far have focused on the lockdown and pre-vaccine period (e.g., Onyeaka et al., 2021; Stockwell et al., 2021). However, studies during other stages of the pandemic's evolution are also needed. This article focuses on data obtained in a Spanish representative sample during the last week of February 2021, about six months after the end of the lockdown period and about two months after the first vaccine injection was administered to highly vulnerable people and essential workers (Spanish Sociological Research center, CIS, 2021).

In this context, the present article aims to identify the impact of Covid-19 fears on somatic health and the relevant paths through which these effects are produced. We concentrate on negative effects of fears, viewing the pandemic as a dramatic event that caused harm in the population. This study contributes to previous knowledge in at least two ways. First, we pay attention to the underlying mechanisms linking Covid-19 fears to somatic problems. Specifically, two potential mechanisms are tested: a) ruminations about the problems associated with Covid-19; and b) psychological distress as a possible reaction to fears. Testing the underlying mechanisms helps to enhance the maturity of science because it clarifies the process, going beyond merely connecting two variables (i.e., fears and somatic problems) (Hayes, 2012). Second, we concentrate on fear as a future-oriented emotion. Humans can engage in affective travelling (Van Dijk et al., 2012), which includes experiencing anticipatory emotions today based on events that could happen in the future (Loewenstein, 1987). Although scholars call for more research on future-oriented emotions (Baumgartner et al., 2008; Seibel et al., 2020), studies on this topic are still limited. We address this call by considering fear as the prototypical anticipatory negative emotion that is experienced in the present but based on the prospect of an undesired event in the future (Baumgartner et al., 2008). This emotion is especially relevant during the Covid-19 pandemic due to uncertainty about the future and the virus's capacity to create health, financial, and social problems (Iob et al., 2022).

### Covid-19 fears and health

Fear, in a generic way, is an adaptive emotion that is fundamental for survival. It involves biological processes that make human beings anticipate risky future events, triggering mechanisms to respond to those potential threats. Specific fears related to Covid-19 may play a functional role when they trigger protective behaviors that prevent contagion and reduce risks. Therefore, fear is a critical factor in understanding individuals’ response to a threat (Witte & Allen, 2000). However, when it is chronic or disproportionate, fear can be harmful, and it may become a key component in the development of mental disorders (García, 2017). Experiencing strong Covid-19 fears can hamper individuals’ health, given the disruptive features of the pandemic, the uncertainty produced, and the social alarm, which is often increased by the media (Chand et al., 2020). Moreover, Covid-19 infection may also produce neurological disorders leading to anxiety through several brain alterations (neurons, glial cells, and/or brain vasculature) (Awogbindin, et al., 2021).

In a recent meta-analysis, Şimşir et al. (2021) found that fear of Covid-19, measured with the FVC-19S scale (Ahorsu et al., 2020), was related to anxiety, stress, and depression. Koçak et al. (2021) also found a significant relationship between Covid-19 fear and stress, anxiety, and depression in the Turkish general population. These studies considered a general measure of Covid-19 fears, although other studies have shown the existence of distinguishable fear facets that may differ in their relations with health and other outcomes (e.g., Rodríguez-Rey et al., 2020; Sandín et al., 2020).

In March 2020, Rodríguez-Rey et al., (2020) identified the following facets in a Spanish sample: (1) health care workers without the capacity to diagnose and treat the coronavirus; (2) a loved one being infected by coronavirus; (3) a shortage of food or health products; (4) insufficient measures taken by the government to control the pandemic; (5) the economic impact of the pandemic; (6) the situation of collective nervousness; (7) not knowing when this crisis is going to end; and (8) their psychological state during the crisis.

Sandín et al., (2020) developed an 18-item scale (FCS) to assess fears during the lockdown period in Spain. They distinguished: (1) fear of infection, disease, and death; (2) fear of scarcity of basic consumer products; (3) fear of social isolation; and (4) fears related to work/income. One year later, the Spanish Sociological Research center launched the "Spaniards’ mental health during the Covid-19 pandemic Survey" (CIS, 2021), which included a modified version of the FCS. The analysis of these data showed the following factors (Peiró et al. 2022): (1) personal health-related fear; (2) fear related to loved ones’ health; (3) economic and employment loss-related fear; (4) and social-related fear. Distinguishing different facets of Covid-19 fear has been useful because, to a certain extent, the facets differ in their vulnerability factors and, especially, in their protective factors. As Sandin and colleagues (2020) pointed out, “income level, work outside the home, and having a private garden predicted several fear types. Age predicts fear of social isolation. The only protective factor that predicts fear of infection/disease/death is positive affect, with weak predictive power” (p. 9). Studies that explore the relations between Covid-19 fears and somatic health problems are scarce (Liu et al., 2020), and it represents an important gap because somatic health problems have been found in the general population during the Covid-19 outbreak. Specifically, data have shown that moderate to high anxiety levels in Covid-19 situations were significantly associated with general somatic symptoms and, particularly, with gastrointestinal and fatigue symptoms (Shevlin et.al., 2020). In fact, somatization has been related to mental disorders during the pandemic (Huang et al., 2020; Saccomanno et al., 2020). However, until now, it has hardly been related to Covid-19 fears (Liu et al., 2020).

This lack of research connecting Covid-19 fears to somatic problems is surprising, given the affective nature of fear and its implications for health. As mentioned above, fear is an anticipatory, future-oriented emotion (Baumgartner et al., 2008). That is, it is experienced today but based on the anticipation of future events (e.g., health problems if infected). Therefore, it is a response to uncertainty (Jordan et al., 2020). According to Uncertainty Management Theory, people want to “feel certain about their world and their place within it” (Van den Bos & Lind, 2002, p. 5). Uncertainty related to important life changes that question the individual's security is associated with a decline in physical health (Maurier & Northcott, 2000; Nelson et al., 1995). Similarly, fear associated with the pandemic could contribute to poor physical health because this emotion reflects a high level of uncertainty about the future, where the status quo and security of the person are challenged. Although this somatization process is complex, and we describe it in greater detail in the following sections, it is reasonable to propose a general relationship between Covid-19 fears and somatic problems.

*H1: The Covid-19 fear factors are positively related to somatic problems.*

### A dual-path model through which Covid-19 fears may impact somatic problems

**Fig. 1:**
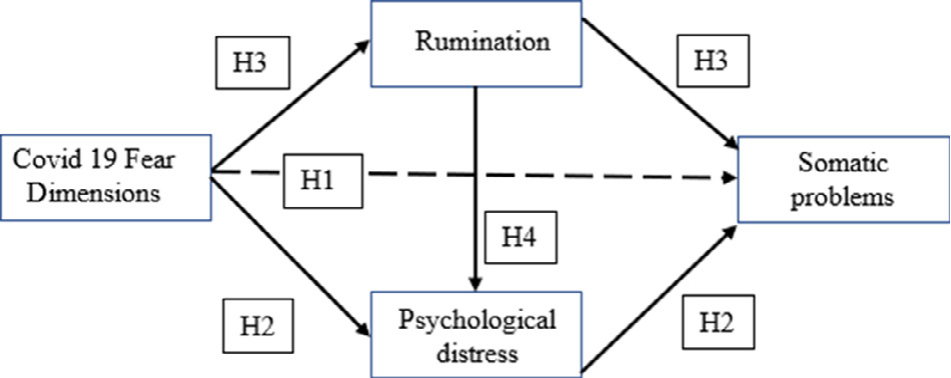
Two-path model of Covid-19 fears’ effects on somatic problems.

Baumeister et al., (2007) suggested that emotions, including fear, contribute to cognitive processes, such as feedback, anticipation, and reflection, that may lead to behaviors and their outcomes. These authors referred to the dual process theory of emotional phenomena, considering both the automatic affective reactions and the full conscious emotions. As they pointed out, “maybe conscious emotion is inextricably intertwined with cognition, whereas automatically affective reactions require nothing more than a perception and an association” (p.168) (see Medrano et al., 2016 for a dual-path model of anxiety). We think the dual-path model from fears to healing is a promising one to integrate the ways Covid-19 fears can have an impact on somatization (Fig. 1).

### The automatic mediation mechanism through psychological distress

The association between fear and psychological distress –defined as a state of emotional suffering, with symptoms such as depression and anxiety (Mirowsky & Ross, 2002)– has often been found, especially during emergency and highly uncertain situations, including the Covid-19 pandemic. Rodríguez-Rey et al., (2020), in a study carried out immediately after the lockdown in Spain, found that the concerns assessed (health, economic, social, etc.) were significantly associated with psychological impact, stress, anxiety, and depression. Other studies have also shown that Covid-19 fears, often measured with the FCV-19 scale (Ahorsu et al., 2020), are positively related to anxiety and depression (Sánchez-Teruel & Bello 2021) and mood disorders (Gao et al., 2020). Suhail et al. (2021) also found a positive correlation between Covid-19 fears and anxiety and depression. Interestingly, this is one of the few studies that analyzes somatic symptoms, and the findings revealed a positive correlation with Covid-19 fears that was stronger when social support was lower. These authors also describe a positive correlation between anxiety and depression and somatic symptoms. Some additional evidence has been obtained on the relations between psychological distress and somatic complaints. Huang et al., 2020 showed that, during the Covid-19 epidemic, anxiety, insomnia, and somatic symptoms were closely related in the general population. Saccomanno et al., (2020) found that perceived stress was strongly correlated with temporomandibular disorders (characterized by depressive symptoms, stress, and painful symptoms).

Taking into account the evidence reviewed and the dual process theory (Baumeister et al., 2007), it is reasonable to expect an automatic somatization process where Covid-19 fear is related to somatic problems through psychological distress. This psychological distress is considered an automatic reaction to fear that, without the intervention of more deliberative regulation or processing, is related to somatic problems.

*H2: Psychological distress mediates the positive relationship between* Covid-19 *fears and somatic problems.*

### The cognitive mediation mechanism through rumination

The second path through which Covid-19 fears might impact somatic problems is a reflexive and cognitive one: rumination. Whereas the former path was more automatic and subconscious, this path involves heavily conscious cognitive processes. Negative rumination has been defined as repetitive thoughts about a negative event (Weigelt et al., 2019) and a tendency to become obsessive about threats and problems. Some authors (Ye et al., 2020) have argued that a more target-specific approach is needed to better capture the cognitive responses to Covid-19. Thus, they studied ruminative tendencies specifically related to the events surrounding Covid-19.

A few studies have shown that persistent fears may lead individuals to a vicious cycle of worry and rumination. Liu et al. (2018) found this relationship in a sample of individuals with previous cancer-related experience. Satici et al. (2020) found that ruminations were significantly correlated with intolerance of uncertainty and Covid-19 fear. Moreover, some evidence was found about the relations between ruminations and somatic health (Verkuil et al., 2010), and more specifically during the pandemic, some somatic complaints such as fatigue (Ye et al., 2020). Thus, based on the aforementioned arguments and considering the dual process theory, we formulate the following hypothesis:

*H3. Ruminations mediate the positive relationship between* Covid-19 *fears and somatic problems.*

**The sequential mediation of rumination and psychological distress between pandemic fears and somatic health problems.**

There is evidence that shows that rumination is connected to psychological distress and if this is the case, a sequential mechanism could mediate Covid-19 fears relation to somatic problems. Some studies have found that processes such as rumination play a role in the psychological distress (Aldao, 2012), and Satici et al. (2020) indicated that rumination increases negative psychological wellbeing in traumatic and uncertain situations. Specifically, during the Covid-19 pandemic, Jamshaid et al. (2020) showed that ruminative thoughts have a negative effect on mental health or psychological distress. In a similar way, Ye et al., (2020) also pointed out that people with a stronger ruminative response style showed more negative emotions and depressive symptoms. Other studies observed that, after the effect of the Covid-19 pandemic, somatic symptoms increased both in healthy patients and in patients with mood disorders, but the prevalence of somatic symptoms was significantly higher in people with mood disorders (Shahini et al., 2021). Taking into account the evidence of this sequential mediation path, in this study, we test the following hypothesis:

*H4* Ruminations mediate the relationship between Covid-19 fears and psychological distress, and psychological distress in turn leads to somatic problems

Therefore, the present article aims to identify the impact of Covid-19 fears on somatic problems (hypothesis 1; H1) and the paths through which this effect is produced: 1) a more automatic process where Covid-19 fear is related to somatic problems through psychological distress (hypothesis 2; H2); 2) a more deliberative process where cognitive rumination about Covid-19 plays a mediator role (hypothesis 3; H3); and 3) a more elaborated process where both rumination and psychological distress describe a mediation sequence (hypothesis 4; H4).

## Material and methods

### Sample and procedure

In the present study, we used data from the “Spaniards’ mental health during the Covid-19 pandemic Survey”, carried out by the Spanish Sociological Research center (CIS) (2021). Data collection took place in February 2021. To contextualize this data collection, it is important to note that the second wave due to the previous Christmas holidays had just ended, and vaccination had just begun in January. The survey was conducted through a computer-assisted telephone interview The sample was composed of 3083 adults from 1080 Spanish municipalities. Almost 51% of the sample was women, and the participants ranged in age from 18 to 98 years old (M = 50.82; *SD* = 16.82).

### Measures

The “Spaniards’ mental health during the Covid-19 pandemic Survey” (SMHC-19S) assessed behaviors, experiences, and consequences of the Covid-19 pandemic. For the present research, we specifically focused on Covid-19 fears, ruminations, psychological distress, and somatic problems. The measures used in this survey were adapted from a previous study carried on by Sandín et al., (2020), as one of the authors participated in the design of the questionnaire of the SMHC-19S. Some items were modified and a few more were added. Then, in the present study we have computed a number of psychometric analyses to confirm the reliability and validity of every variable used. In what follows we briefly describe the psychometric properties of each variable used.

Covid-19 *fears* were measured with a 15-item scale, asking participants to evaluate the degree to which they have experienced fears related to Covid-19 (see Sandín et al., 2020). The response scale ranged from 1 (very much) to 5 (not at all); however, it was inverted to facilitate the reader's understanding (thus, 1 –not at all-; 5 - very much-). Exploratory factor analyses and confirmatory factor analyses results showed that the expected four fear factors exist in this scale (Root Mean Square Error Approximation; RMSEA = 0.052; Comparative Fit Index; CFI = 0.965; Tucker-Lewis Index; TLI = 0.955; Root Mean Square Residual; SRMR = 0.029) (Peiró et al., in preparation): four items were related to *personal health-related fears* (sample item: “fear of dying from Covid-19″); four items focused on *fears related to relatives’ or loved ones’ health* (sample item: “fear that a family member or loved one might die from Covid-19″); three items referred to *economic/employment-related fears* (sample item: “fear of losing income”); and four items measured *social-related fears* (sample item: “fear that society will no longer be the same as before”). Cronbach alpha in this study ranged from 0.76 to 0.84.

*Ruminations about* Covid-19 were measured with a seven-item scale (sample item: “Could you tell me how many times you have had unwanted unpleasant thoughts or memories about the coronavirus and its consequences?”). The scale included some items referring to negative alterations in cognitions from the posttraumatic stress measure (See Blevis et al., 2015; Sandín et al., 2020). The response scale ranged from 1 (A lot of times) to 4 (None or almost none); however, it was inverted to facilitate the reader's understanding (thus, 1 –none or almost none-; 4– a lot of times-). The Cronbach *a* in this study for this scale was 0.81.

*Psychological distress* was measured with a nine-item scale (sample item: “from the beginning of the Covid-19 pandemic and until now, could you tell me how many times you have felt very sad or depressed?”). The response scale ranged from 1 (A lot of times) to 4 (None or almost none); however, it was inverted to facilitate the reader's understanding (thus, 1 –none or almost none-; 5 – a lot of times-). The Cronbach *a* for the scale in this study was 0.91.

*Somatic problems* were measured with a 14-item scale (sample item: “From the beginning of the Covid-19 pandemic and until now, have you felt bad about having a stomachache?”). Participants should answer “yes” or “no” for each item. Then, a cumulative index was computed that ranged from 0 (no physical symptoms) to 14 (all the physical symptoms on the scale).

Finally, we controlled the effect of *having a chronic long-term illness not related to* Covid-19*.* The response scale ranged from 1 (yes) to 2 (no); however, it was inverted to facilitate the reader's understanding (thus, 0 –no-; 1 –yes-).

### Data analysis

First, means, standard deviations, and correlations (Pearson) were computed using SPSS v.26. Second, we carried out Structural Equation Modeling (SEM) to determine the relations between the variables of interest. To this end, we used MPlus software (Muthén & Muthén, 1998–2015). To test the significance of the indirect effects, we produced confidence intervals using the Monte Carlo Method for Assessing Mediation (Preacher & Selig, 2012) with 20,000 repetitions.

In order to assess the model fit, we examined the RMSEA, CFI, TLI, and SRMR goodness of fit statistics. For the ML method, a cutoff value close to 0.08 for RMSEA typically indicates a reasonable fit (Little, 2013); a cutoff value greater than 0.90 for CFI and TLI typically indicates an acceptable fit to the data (Little, 2013); and a cutoff value of less than 0.08 for SRMR indicates a relatively good fit between the hypothesized model and the observed data (Hu & Bentler, 1999).

## Results

**Fig. 2:**
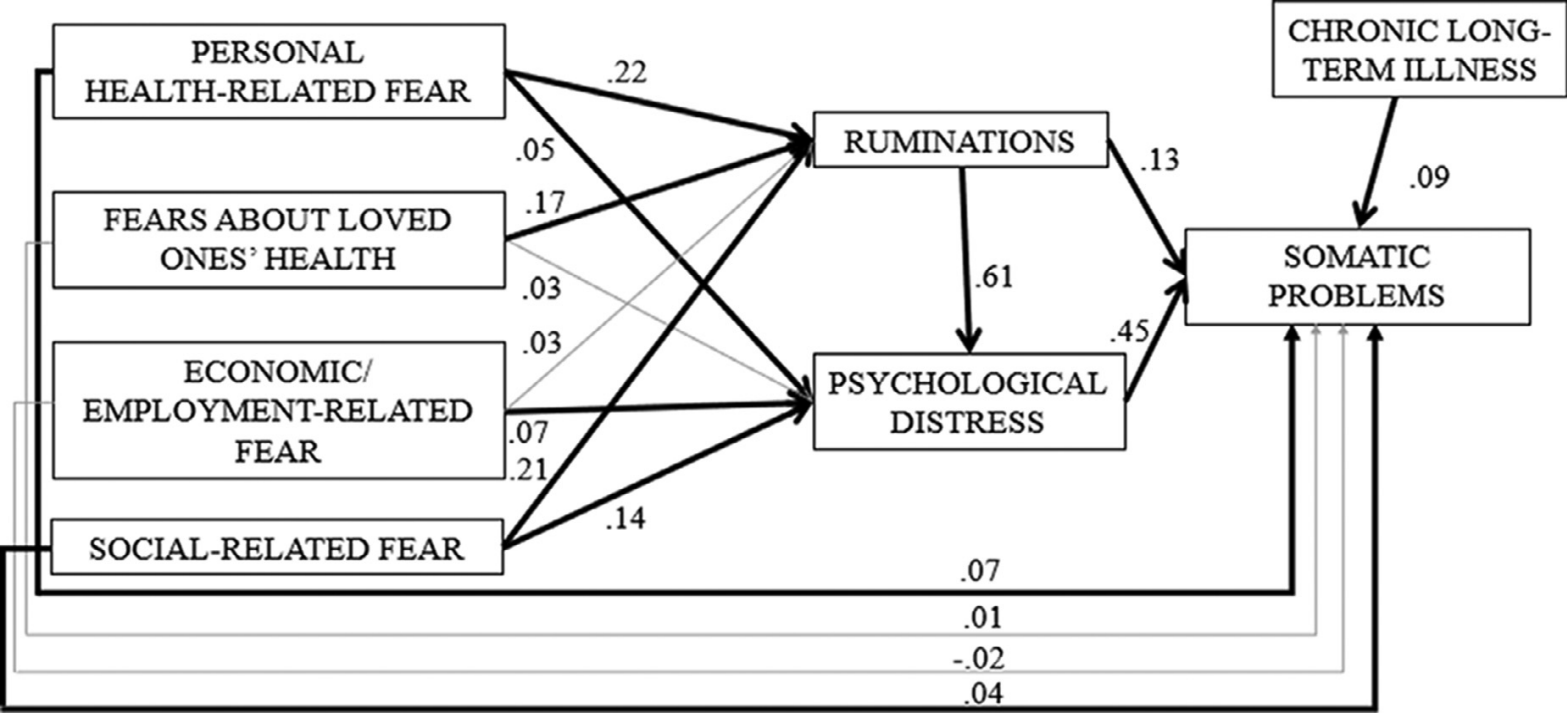
SEM results.

Means, standard deviations, and correlations (Pearson) are presented in Table 1. Results of the SEM showed a good fit for the proposed model (RMSEA = 0.017, CFI = 1.000, TLI = 0.997 and SRMR = 0.004). The set of variables in the model explain 37.0% of somatic problems (*p* < .05). Fig. 2 shows the results.

**Table 1:** Means, standard deviations, T tests, and correlations.

	*N* = 3083							
	*M* (SD)	1	2	3	4	5	6	7
1. Personal health-related fears	2.81 (1.13)	-						
2. Fears about Loved ones’ health	3.83 (1.03)	.62*	–					
3. Economic-employment-related fears	2.99 (1.29)	.38*	.40*	–				
4. Social-related fears	3.25 (1.08)	.57*	.58*	.48*	–			
5. Ruminations	1.71 (0.64)	.47*	.45*	.29*	.46*	–		
6. Psychological distress	1.80 (0.73)	.46*	.44*	.35*	.50*	.74*	–	
7. Somatic problems	3.39 (3.19)	.35*	.31*	.25*	.33*	.51*	.60*	–

⁎ *p*<.05.

### Direct effects of Covid-19 fears on somatic problems

Results yield partial support for hypothesis 1, which states that Covid-19 fears are positively related to somatic problems. Results support the direct association between both personal health-related fears (*Est.*= 0.07*; p*< .05) and social-related fears (*Est.*= 0.04*, p*< .05) and somatic problems, but they do not support the direct links between fears about loved ones’ health (*Est.*= 0.01*, p* >0.05) or economic/employment-related fears (*Est.*= −0.02*, p* >0.05) and somatic problems.

Additionally, results show that all the Covid-19 fears (except fears about loved ones’ health) were positively related to psychological distress (*p*< .05). Similarly, all the fear factors (except economic/employment-related fear) were positively related to ruminations (*p*< .05). Moreover, both ruminations and psychological distress were positively linked to somatic problems (*Est.= 0.13, p*< .05 and *Est.*= 0.45*, p*< .05, respectively*)*. Finally, ruminations also showed a significant positive relationship with psychological distress (*Est.*= 0.61*, p*< .05).

### Indirect or mediated effects of Covid-19 on somatic problems

Hypotheses 2, 3, and 4 proposed indirect effects between the pandemic fear dimensions and somatic problems (Table 2). First, hypothesis 2 states that these relations are mediated by psychological distress, but the results did not support this idea because no indirect significant effects were found. Thus, the existence of a more automatic mechanism (based on psychological distress) that connects Covid-19 fears to somatic problems was not identified in this study.

**Table 2:** Indirect effects.

	Confidence intervals
*Pandemic Fears’ positive effects on somatic problems:* *Through psychological distress*	
Personal health-related fear	LL −0.06; UL 0.06
Fears about loved ones’ health	LL −0.04; UL 0.04
Economic/employment-related fear	LL-0.09; UL 0.10
Social-related fear	LL-0.19; UL 0.19
*Through ruminations*	
Personal health-related fear	LL-0.02; UL 0.07
Fears about loved ones’ health	LL −0.01; UL 0.06
Economic/employment-related fear	LL-0.01; UL 0.01
Social-related fear	LL-0.02; UL 0.07
*Through ruminations and psychological distress, in that order*	
Personal health-related fear	LL 0.04; UL 0.09*
Fears about loved ones’ health	LL 0.03; UL 0.07*
Economic/employment-related fear	LL 0.01; UL 0.02*
Social-related fear	LL 0.03; UL 0.08*

⁎ Confidence intervals that do not include zero show significant indirect effects.

Second, results did not provide support for hypothesis 3, which states that the relations between pandemic fears and somatic problems are mediated by rumination. Again, none of the Covid-19 fear factors showed a significant indirect effect on somatic problems through rumination. Thus, the rumination mechanism linking Covid-19 fears to somatic problems was rejected.

Finally, hypothesis 4 states that the relationship between pandemic fears and somatic problems is mediated sequentially by ruminations and psychological distress, in that order. Results support these indirect effects for all the pandemic fear dimensions: personal health-related fear [LL 0.04; UL 0.09]; fears about loved ones’ health [LL 0.03; UL 0.07]; economic/employment-related fear [LL 0.01; UL 0.02]; and social-related fear [LL 0.03; UL 0.08]. Therefore, the path between Covid-19 fears and somatic problems is based on a more complex process involving both rumination and psychological distress.

### Auxiliary analysis: testing alternative processes

Our results are suggesting that Covid-19 fears are the antecedents of a process that leads to somatic problems through ruminations and psychological distress (H4). Although the CIS survey about mental health during Covid-19 allowed us to use data from a big and representative sample in Spain during the pandemic, it was based on a cross-sectional design that prevents us from drawing causal conclusions. That is, other process alternatives are possible where the order of constructs is different.^1^ To examine other alternative processes, we tested as auxiliary analyses three additional models. First, we tested an alternative model where somatic problems are antecedents of Covid-19 fears through ruminations and psychological distress. Experiencing somatic problems could lead to ruminations and psychological distress, which in turn could increase fears related with the possible negative consequences of Covid-19. This alternative model (RMSEA = 0.021; CFI = 1.000; TLI = 0.996; SRMR = 0.007) had worse fit than the proposed model.

Second, we examined an alternative model where ruminations and psychological distress lead to somatic problems through Covid-19 fears. Accordingly, people who experienced ruminations and psychological distress during the pandemic could feel Covid-19 fears, increasing their somatic problems. Again, this alternative model (RMSEA = 0.283; CFI = 0.639; TLI = 0.098; SRMR = 0.115) had worse fit than the proposed model.

Finally, the third alternative model describes a process where ruminations and psychological distress lead to Covid-19 fears through somatic problems. People who experienced ruminations and psychological distress during the pandemic could suffer somatic problems, increasing their fears associated with possible negative consequences of Covid.19. As occurred with the other alternative models, this model also had worse fit (RMSEA = 0.089; CFI = 0.995; TLI = 0.927; SRMR = 0.018) than the proposed one. In sum, all alternative models had worse fit than our proposal. This suggests that, although data from this study is based on a cross-sectional design and it prevents us from drawing causal conclusions, the comparison of the proposed model with other alternative models that had worse fit gives us indications that the sequence that we have proposed (hypothesis 4) could tentatively describe the negative experiences during the pandemic.

## Discussion

This study examined the underlying mechanisms linking Covid-19 fears to somatic problems. In addition to the direct relationship, three proposals were tested: first, a more automatic mechanism with psychological distress as the mediator; second, a more deliberative proposal with cognitive rumination as the mediator; and finally, an extended sequence where rumination and psychological distress jointly played a mediating role. Results supported this latter proposal: rumination mediates the links from Covid-19 fears to psychological distress, and psychological distress in turn is related to somatic problems. Implications of the results are discussed below.

### Theoretical implications

Although previous research has identified possible precursors of somatic problems associated with Covid-19, such as psychological distress (Huang et al., 2020) and ruminations (Ye et al., 2020), there was a need to clarify the process through which somatizations occur in crisis situations such as this pandemic. Our supported sequence (fears – ruminations – psychological distress – somatic problems) contributes to clarifying this issue. Paying attention to underlying mechanisms enhances the maturity of science by going beyond the mere relationship between two variables (Hayes, 2012). Thus, scholars are increasingly interested in the psychosocial processes that potentially explain human behavior (Johnson et al., 2017). Instead of concentrating on the factors that explain the probability that certain results will occur, attention is paid to the variables that act throughout a process that explains the final response (Rahal & Fiedler, 2019). We adopted this strategy to investigate somatic problems during the Covid-19 pandemic. Research has found that the pandemic can lead to somatic symptoms (Shevlin et.al., 2020). However, testing the processes involved allows us to understand plausible routes. In our study, Covid-19 fears produce somatization because they stimulate rumination and psychological distress. This research approach provides a richer understanding of somatic problems in the general population during the pandemic.

Another relevant contribution of the present study is the consideration of Covid-19 fear as a future-oriented emotion. Humans can engage in mental and affective travelling towards the future. This capacity has facilitated survival (Kabadayi & Osvath, 2017) because the person can project him/herself into pre-lived events (Suddendorf & Corballis, 1997). Despite the importance of future-oriented emotions in human life and calls for research in this direction (Baumgartner et al., 2008; Seibel et al., 2020), scholars traditionally pay more attention to emotions associated with events that have already occurred (Ilies & Judge, 2002). This approach has provided meaningful knowledge; however, without considering the future, it is difficult to obtain an adequate understanding of the experiences of the general population in crisis situations such as a pandemic. Fear is an anticipatory emotion that is experienced in the present, but based on future events (Baumgartner et al., 2008). This is particularly relevant when facing possible health, economic, and social problems due to the spread of the virus, and it is congruent with Uncertainty Management Theory (Van den Bos & Lind, 2002). Fear informs us about challenges to our ideas about the world and our place in it. According to our sequence, fear is the starting point that leads to the final response of somatization.

In fact, although the role of Covid-19 fear is strong enough to establish some direct relations with psychological distress and somatic problems (see Figure 2), the mediation through rumination (and subsequent psychological distress) is especially helpful in understanding the process of somatization. Of course, fear can be functional, alerting people to possible negative events in the future and making it easier to prepare to deal with problems. However, when fear turns into repetitive thoughts about a negative event (Weigelt et al., 2019), the person is more likely to experience psychological distress and somatic problems. During the pandemic, many people in the general population may have experienced continuous and obsessive thoughts about the virus and its consequences.

### Practical implications

Today's societies are likely to face crises such as the Covid-19 pandemic (other pandemics, climate crises, wars, etc.) that generate fears and problems in the population. Our findings have relevant implications for practice at the individual and societal level. At the individual level, training people to manage their fears could help. As mentioned above, fears can be functional because they warn the person about possible problems. However, fears are dysfunctional if they become negative obsessive thoughts that produce psychological distress and somatization. This training could be implemented in different ways by considering not only the direct intervention of mental health professionals, but also the indirect participation of relevant actors with credibility (doctors, teachers, etc.) who have direct contact with different groups in the population.

At the societal level, the media and social networks play a crucial role. They can overemphasize bad news, creating exaggerated fears in the population (Lin et al., 2020). Media, governments, authorities, and civil society should organize a communication system that provides accurate information about crises, avoiding unnecessary fears. This practice, based on evidence, could also be encouraged in different contexts (universities, companies, public administrations, schools, NGOs, etc.).

### Limitations

The present study has limitations that provide input for future studies. First, we used a large representative sample in a specific country (Spain), which is a strength of the study, but this design does not allow us to test the generalizability of our findings outside this country. Although fear is a universal emotion, its experience could vary depending on the cultural framework (Ali et al., 2021). Therefore, testing our model in other countries would make it possible to examine its universality. Second, as our study is based on a cross-sectional design, we cannot investigate issues of directionality or causality in the data. Thus, our results only give us tentative indications that the sequence that we have proposed could describe the negative experiences during the pandemic.

Finally, our study concentrates on the prototypical negative future-oriented emotion: fear. This option is reasonable given the problems produced by the pandemic. Nevertheless, considering the prototypical positive future-oriented emotion (hope) (Baumgartner et al., 2008) could provide complementary knowledge. For example, it would be useful to study whether hope about positive changes during the pandemic (e.g., a more compassionate society) can enhance positive affect and reduce somatic problems.

## Conclusions

The current study contributes to previous knowledge by clarifying how Covid-19 fears lead to somatic problems in the general population. Beyond direct relations, our findings support a process where rumination mediates between fear and psychological distress, and psychological distress in turn leads to somatic problems. This process reveals a plausible mechanism in the somatization of health problems during the Covid-19 pandemic.

## Footnotes

1 We are grateful to an anonymous reviewer for pointing out this possibility to us.

## References

[bib0001] Ahorsu D.K., Lin C.Y., Pakpour A.H. (2020). The association between health status and insomnia, mental health, and preventive behaviors: The mediating role of fear of COVID-19. Gerontology & Geriatric Medicine.

[bib0002] Aldao A. (2012). Emotion regulation strategies as transdiagnostic processes: A closer look at the invariance of their form and function. Revista de Psicopatología y Psicología Clínica.

[bib0003] Ali M., Uddin Z., Banik P.C., Hegazy F.A., Zaman S., Ambia A. (2021). Knowledge, attitude, practice, and fear of COVID-19: An online-based cross-cultural study. International Journal of Mental Health and Addiction.

[bib0005] Baumeister R.F., Vohs K.D., Nathan DeWall C., Zhang L. (2007). How emotion shapes behavior: Feedback, anticipation, and reflection, rather than direct causation. Personality and Social Psychology Review.

[bib0006] Baumgartner H., Pieters R., Bagozzi R.P. (2008). Future-oriented emotions: Conceptualization and behavioral effects. European Journal of Social Psychology.

[bib0008] Bueno-Notivol J., Gracia-García P., Olaya B., Lasheras I., López-Antón R., Santabárbara J. (2021). Prevalence of depression during the COVID-19 outbreak: A meta-analysis of community-based studies. International Journal of Clinical and Health Psychology.

[bib0009] Chand D., Verma T., Verma R., Taneja A., Jena S. (2020). COVID-19: Impacts of quarantine on mental health and stress. Indian Journal of Public Health Research & Development.

[bib0010] Gao J., Zheng P., Jia Y., Chen H., Mao Y., Chen S. (2020). Mental health problems and social media exposure during COVID-19 outbreak. PLoS ONE.

[bib0011] García R. (2017). Neurobiology of fear and specific phobias. Learning & Memory.

[bib0012] Green E.C., Witte K. (2006). Can fear arousal in public health campaigns contribute to the decline of HIV prevalence?. Journal of Health Communication.

[bib0013] Hayes, A.F. (2012). PROCESS: A versatile computational tool for observed variable mediation, moderation, and conditional process modeling. Retrieved from http://www.afhayes.com/public/process2012.pdf

[bib0014] Hu L.T., Bentler P.M. (1999). Cutoff criteria for fit indexes in covariance structure analysis: Conventional criteria versus new alternatives. Structural Equation Modeling: A Multidisciplinary Journal.

[bib0015] Huang Y., Wang Y., Zeng L., Yang J., Song X., Rao W. (2020). Prevalence and correlation of anxiety, insomnia and somatic symptoms in a Chinese population during the Covid-19 epidemic. Front Psychiatry.

[bib0016] Ilies R., Judge T.A. (2002). Understanding the dynamic relations among personality, mood and job satisfaction: A field experience sampling study. Organizational Behavior and Human Decision Processes.

[bib0017] Iob E., Steptoe A., Zaninotto P. (2022). Mental health, financial, and social outcomes among older adults with probable COVID-19 infection: A longitudinal cohort study. Proceedings of the National Academy of Sciences.

[bib0018] Jamshaid S., Malik N.I., Haider A.A., Jamshed K., Jamshad S. (2020). Proceedings of the international joint conference on arts and humanities (IJCAH).

[bib0019] Johnson D.J., Hopwood C.J., Cesario J., Pleskac T.J. (2017). Advancing research on cognitive processes in social and personality psychology: A hierarchical drift diffusion model primer. Social Psychological and Personality Science.

[bib0020] Jordan P., Troth A., Ashkanasy N., Humphrey R. (2020). The cambridge handbook of workplace affect.

[bib0021] Kabadayi M., Osvath (2017). Ravens parallel great apes in flexible planning for tool-use and bartering. Science.

[bib0022] Koçak O., Koçak Ö.E., Younis M.Z. (2021). The psychological consequences of COVID-19 fear and the moderator effects of individuals’ underlying illness and witnessing infected friends and family. International Journal of Environmental Research and Public Health.

[bib0023] Lin, C.Y., .Broström, A., Griffiths, M.D., .& Pakpour, A.H. (2020). Investigating mediated effects of fear of COVID-19 and COVID-19 misunderstanding in the association between problematic social media use, psychological distress, and insomnia. Internet interventions, 21, 100345. 10.1016/j.invent.2020.100345 10.1016/j.invent.2020.100345 PMC7449889 32868992

[bib0024] Little T.D. (2013).

[bib0025] Liu J., Peh C.X., Simard S., Griva K., Mahendran R. (2018). Beyond the fear that lingers: The interaction between fear of cancer recurrence and rumination in relation to depression and anxiety symptoms. Journal of Psychosomatic Research.

[bib0026] Liu S., Liu Y., Liu Y. (2020). Somatic symptoms and concern regarding COVID-19 among Chinese college and primary school students: A cross-sectional survey. Psychiatry Research.

[bib0027] Loewenstein G. (1987). Anticipation and the valuation of delayed consumption. The Economic Journal.

[bib0028] Luo F., Ghanei Gheshlagh R., Dalvand S., Saedmoucheshi S., Li Q. (2021). Systematic review and meta-analysis of fear of COVID-19. Frontiers in Psychology.

[bib0029] Maurier W.L., Northcott H.C. (2000). Job uncertainty and health status for nurses during restructuring of health care in Alberta. Western Journal of Nursing Research.

[bib0030] Medrano L.A., Muñoz-Navarro R., Cano-Vindel A. (2016). Procesos cognitivos y regulación emocional: Aportes desde una aproximación psicoevolucionista. Ansiedad y estrés.

[bib0031] Mertens G., Gerritsen L., Duijndam S., Salemink E., Engelhard I.M. (2020). Fear of the coronavirus (COVID-19): Predictors in an online study conducted in March 2020. Journal of Anxiety Disorders.

[bib0032] Mirowsky J., Ross C.E. (2002). Selecting outcomes for the sociology of mental health: Issues of measurement and dimensionality. Journal of Health and Social Behavior.

[bib0033] Morman M.T. (2000). The influence of fear appeals, message design, and masculinity on men's motivation to perform the testicular self-exam. Journal of Applied Communication Research.

[bib0034] Muthén B., Muthén L. (1998).

[bib0035] Nelson A., Cooper C.L., Jackson P.R. (1995). Uncertainty amidst change: The impact of privatization on employee job satisfaction and well-being. Journal of Occupational and Organizational Psychology.

[bib0036] Onyeaka H., Anumudu C.K., Al-Sharify Z.T., Egele-Godswill E., Mbaegbu P. (2021). COVID-19 pandemic: A review of the global lockdown and its far-reaching effects. Science Progress.

[bib0037] Peiró, J.M., .Soriano, A., Luque, A., & Martínez-Tur, V. (2022). (in preparation): The assessment of fears of the Covid-19 Pandemic in Spain: A study on the Fears of Coronavirus Scale (FCS).

[bib0038] Preacher K.J., Selig J.P. (2012). Advantages of Monte Carlo confidence intervals for indirect effects. Communication Methods and Measures.

[bib0039] Rahal R., Fiedler S. (2019). Understanding cognitive and affective mechanisms in social psychology through eye-tracking. Journal of Experimental Social Psychology.

[bib0041] Rodríguez-Rey R., Garrido-Hernansaiz H., Collado S. (2020). Psychological impact of COVID-19 in Spain: Early data report. Psychological Trauma: Theory, Research, Practice, and Policy.

[bib0042] Saccomanno S., Bernabei M., Scoppa F., Pirino A., Mastrapasqua R., Visco M.A. (2020). Coronavirus lockdown as a major life stressor: Does it affect TMD symptoms?. International Journal of Environmental Research and Public Health.

[bib0043] Sánchez-Teruel D., Bello M.A.R. (2021). Escala de miedo al COVID-19 (FCV-19S): Propiedades psicométricas e invariabilidad de la medida en la versión española. Actas Españolas de Psiquiatría.

[bib0044] Sandín B., Valiente R.M., García-Escalera J., Chorot P. (2020). Impacto psicológico de la pandemia de COVID-19: Efectos negativos y positivos en población española asociados al periodo de confinamiento nacional. Revista de Psicopatología y Psicología Clínica.

[bib0045] Satici B., Saricali M., Satici S.A., Griffiths M.D. (2020). Intolerance of uncertainty and mental wellbeing: Serial mediation by rumination and fear of COVID-19. International Journal of Mental Health and Addiction.

[bib0046] Seibel S., Volmer J., Syrek C.J. (2020). Get a taste of your leisure time: The relationship between leisure thoughts, pleasant anticipation, and work engagement. European Journal of Work and Organizational Psychology.

[bib0047] Shahini N., Salimi Z., Javan M., Kamkar M. (2021). Evaluation of the Covid-19 pandemic effect on the development of somatic symptoms in patients with mood disorders: A case-control study. New Microbes and New Infections.

[bib0048] Shevlin M., Nolan E., Owczarek M., McBride O., Murphy J., Gibson J. (2020). COVID-19 related anxiety predicts somatic symptoms in the UK population. British Journal of Health Psychology.

[bib0049] Şimşir Z., Koç H., Seki T., Griffiths M.D. (2021). The relationship between fear of COVID-19 and mental health problems: A meta-analysis. Death Studies.

[bib0050] Spanish Sociological Research Center (CIS (2021). https://www.cis.es/cis/export/sites/default/-Archivos/Marginales/3300_3319/3312/es3312mar.pdf.

[bib0051] Stockwell S., Trott M., Tully M., Shin J., Barnett Y., Butler L. (2021). Changes in physical activity and sedentary behaviours from before to during the COVID-19 pandemic lockdown: A systematic review. BMJ Open Sport & Exercise Medicine.

[bib0052] Suddendorf T., Corballis M.C. (1997). Mental time travel and the evolution of the human mind. Genetic, Social, and General Psychology Monographs.

[bib0053] Suhail A., Iqbal N., Smith J. (2021). Lived experiences of Indian Youth amid COVID-19 crisis: An interpretative phenomenological analysis. International Journal of Social Psychiatry.

[bib0054] van den Bos K., Lind E.A. (2002). Advances in experimental social psychology.

[bib0055] Van Dijk L.F., Van Dillen E.C., Seip M., Rotteveel (2012). Emotional time travel: Emotion regulation and the overestimation of future anger and sadness. European Journal of Social Psychology.

[bib0056] Verkuil B., Brosschot J.F., Gebhardt W.A., Thayer J.F. (2010). When worries make you sick: A review of perseverative cognition, the default stress response and somatic health. Journal of Experimental Psychopathology.

[bib0057] Weigelt O., Gierer P., Syrek C.J. (2019). My mind is working overtime—towards an integrative perspective of psychological detachment, work-related rumination, and work reflection. International Journal of Environmental Research and Public Health.

[bib0058] Witte K., Allen M. (2000). A meta-analysis of fear appeals: Implications for effective public health campaigns. Health Education & Behavior.

[bib0059] Ye B., Zhou X., Im H., Liu M., Wang X.Q., Yang Q. (2020). Epidemic rumination and resilience on college students’ depressive symptoms during the COVID-19 pandemic: The mediating role of fatigue. Front Public Health.

[bib0060] Zhang H., Li W., Li H., Zhang C., Luo J., Zhu Y. (2021). Prevalence and dynamic features of psychological issues among Chinese healthcare workers during the COVID-19 pandemic: A systematic review and cumulative meta-analysis. General Psychiatry.

